# Is sterile supply department meant for sterilization or adding more trouble?

**DOI:** 10.4103/0019-5049.60509

**Published:** 2010

**Authors:** Kiran Dasari

**Affiliations:** Hurstwood Park Neurological Centre, Haywards Heath, UK

**Figure 1:**
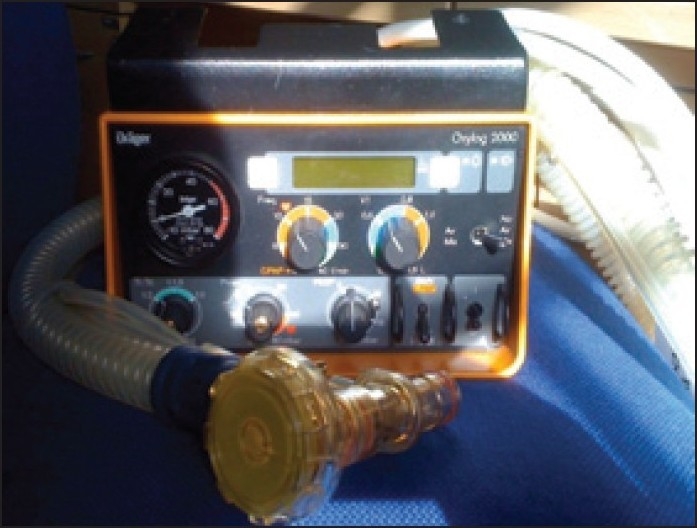
Drager Oxylog 2000 ventilator

**Figure 2:**
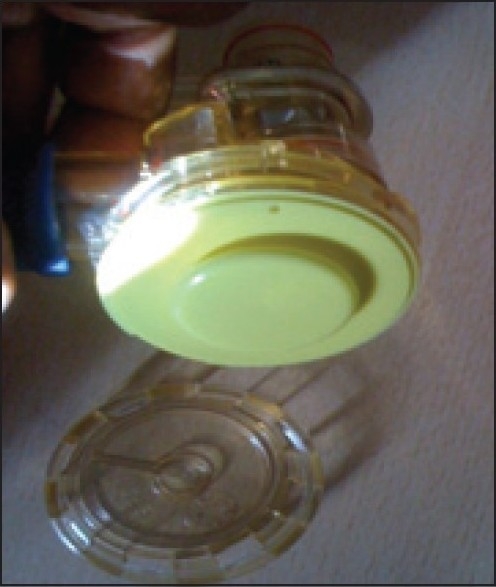
Incorrectly positioned silicone diaphragm

**Figure 3:**
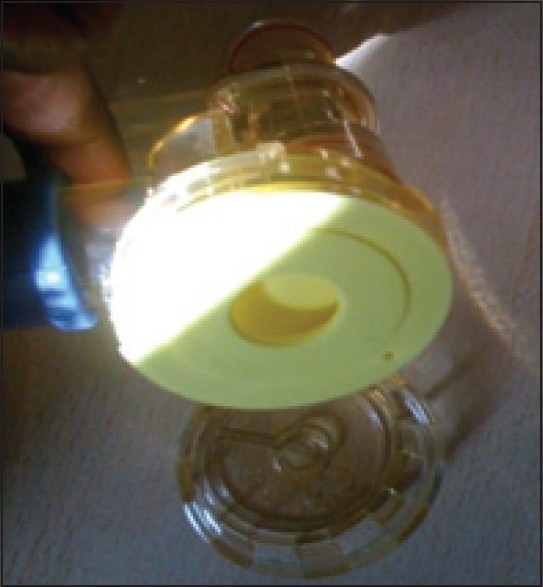
Correctly positioned silicone diaphragm

Sir,

We would like to highlight the importance of checking the portable ventilator before usage for each patient. The Oxylog 2000 ventilator [Figure 1] is being used in our neurointensive care for ventilation during CT scans and patient transfers. A 24-year-old male patient was due for an emergency computerised tomography (CT) scan following head injury. After the ventilator was connected to the oxygen cylinder and the test lung, it failed to ventilate. The connections were intact, ventilator settings were appropriate, and oxygen cylinder was full. There were no leaks anywhere in the circuit. The other similar ventilator was tested and it was functioning well. After getting the CT scan done with the functioning ventilator, we tried to identify the problem with the non functioning ventilator. On careful inspection, the silicone diaphragm in the ventilator valve was found to be inserted in an incorrect position[1] [Figure 2]. Apparently, this was done by the sterile services department during sterilisation. Once it was correctly inserted, [Figure 3] the ventilator functioned perfectly well. We strongly recommend this additional checking of silicone diaphragm positioning in case of routine ventilator troubleshoot.
